# Psychological wellbeing with music therapy: the moderating role of health awareness, and strategic health management in post Covid-19 era

**DOI:** 10.1186/s40359-024-01845-z

**Published:** 2024-06-18

**Authors:** Yang Liu

**Affiliations:** 1 School of Architecture and Art Design, Inner Mongolia University of Science and Technology, Baotou, China; 2 Philippine Christian University, Manila, Philippines

**Keywords:** Psychological well-being, Music therapy, Health awareness, Strategic health management

## Abstract

**Background:**

Psychological problems are common among the people of every community. These psychological issues are leading people to mental health issues. Human well-being is required to be improved appropriately for the better health of the public. The objective of this research is to determine the influence of music therapy on the sustainable psychological well-being of the Chinese community. Furthermore, this research determines that moderating role of health awareness and strategic health management between music therapy and sustainable psychological well-being.

**Method:**

The research used a sample of 384 collected with a random sampling method. For data collection, a cross-sectional method was adopted to collect data on a Likert scale questionnaire. The Health Awareness Scale, Music Therapy Scale, Sustainable Health Management Scale and Sustainable Psychological Wellbeing Scale was used in this research.

**Results:**

The findings of the research highlighted that there is a significant and positive influence of music therapy on the sustainable psychological well-being of the Chinese community. This research also concluded that there is a significant and positive moderating role of health awareness and strategic health management between music therapy and sustainable psychological well-being. The findings of this research are new and novel in the literature on psychological well-being.

**Conclusion:**

This research has some theoretical and practical implications to advance the literature and practice for sustainable psychological well-being respectively. In clinical practice, music therapy can be effectively used to improve the psychological well-being of individuals with sustainability. The information related to health awareness and practice for strategic health management is also necessary for the clinical patients to improve their psychological well-being.

**Supplementary Information:**

The online version contains supplementary material available at 10.1186/s40359-024-01845-z.

## Introduction

Mental issues are common in modern society because people are depressed and they have different concerns. Indeed, people in the past also faced mental health-related issues that were disturbing for them to improve their behavior and learning [16]. The better way to deal with people’s mental issues is to provide them with appropriate treatment on time. Mental health issues should be treated on time because when these issues would not treat on time, people may face significant problems in their life [37]. The behavior of mentally ill people is different from that of normal people, and this distinction is disturbing for people. The available resource for health improvement is required for people to get better treatment. Different kinds of clinical treatment are available for those people who are mentally not well [1]. However, they are required to take interesting initiatives to improve their health in a better way. The mental health of the public helps them to perform better in their routine life.

Indeed, there are different clinical treatments for people who are facing mental health issues [34]. However, music therapy is an emerging concept in Western countries regarding mental health issues. People who are facing mental health issues are required to get better clinical treatment to improve their health status [20]. However, many patients don’t rely on the significance and outcomes of mental health with clinical treatment. The stability of mental health issues for the public is required to be improved over time [3]. The success of people towards mental health stability helps them to enjoy life. The Chinese community is in large numbers in the world, and there are many mental health issues reported in this community. People who are facing mental health issues are required to get clinical treatment effectively. However, it is also noted that people have very limited information related to mental health issues.

 [35] reported that physical therapy helps to improve the psychological well-being of people. Psychosocial well-being is defined as the state of mental, emotional, and social health of an individual. It is a broad concept that encompasses various aspects of human life, including personal growth, happiness, life satisfaction, self-esteem, social functioning, and a sense of purpose in life. The study [19] highlighted that people who do not have effective treatment, face problems in the long term. However [7], reported that the mental health issues for the people should be maintained in time to ensure that their performance would be improved [12]. asserted that therapy with modern music can guarantee the successful mental treatment of people [5]. also highlighted that music therapy should be used appropriately to improve the health behavior of the people. In the meanwhile [25], asserted that music therapy has been less discussed from the perspective of sustainable psychological well-being, as earlier studies only focused on the role of music therapy in mental health improvement briefly. Furthermore, health awareness and strategic health management are critical factors but are not discussed widely in the existing body of literature.

Due to workload, social pressure, following the norms, and psychological illness, the Chinese community face problems related to psychological well-being. It is problematic for them to make good decision in bad psychological condition. On the other hand, health management is necessary for the public to improve their mental and physical health. The appropriate mental health is required for the individuals to perform their tasks in the better way. However, the individuals who are good in managing their health, it is appropriate for them to improve their health strategically. Hence, health management is considered as critical factor for improving mental health and psychological well-being. Therefore, the objective of this research is to determine the influence of music therapy on the sustainable psychological well-being of the Chinese community. Furthermore, this research determines that moderating role of health awareness and strategic health management between music therapy and sustainable psychological well-being. The research used a sample of 384 collected with a random sampling method. For data collection, the cross-sectional method was adopted to collect data on the Likert scale questionnaire. The findings of this research are new and novel in the literature on psychological well-being. This research has some theoretical and practical implications to advance the literature and practice for sustainable psychological well-being respectively. This research also has some limitations that are required to be addressed by future studies.

## Review of literature

### Theoretical underpinning

Psychodynamics theory explains that the relationship between human behavior and emotions is influenced by their psychology [6]. It is necessary to influence human behavior for the right working, but this behavior is reported to be improved with psychological improvement. Human emotions are key to their performance and way of sustainability. The behavior studies highlight that human behavior is required to be improved. In this way, Psychodynamics theory significantly highlights that the underlying human emotions can be triggered with the psychological empowerment that is necessary for advancing their behavior in the right direction [40]. Access to human behavior and emotions for better psychological well-being is necessary for their emotional treatment. In modern times, different kinds of psychological treatments are recommended for human behavior to influence their understanding in the right way. Furthermore, Psychodynamics theory focuses on human emotions and other influencing drivers that are supporting to improve human behavior in a significant way. The right to work for human emotions can improve human being performance for better living standards. Access to human behavior and emotions by way of influencing drivers can improve human emotions in the right direction. By the underpinning of Psychodynamics theory, this research has considered music therapy as the influencing factor for sustainable psychological well-being as the dependent variable.

### Hypotheses development

Music therapy is considered one of the critical ways to improve human behavior and emotions. Human emotions are positively and significantly influenced when human behavior is different [39]. The advancement of human behavior and psychology is helpful for them to perform well in different situations. There are different kinds of cases for people who are struggling with psychological well-being, and the people are required to improve their behavior and learning. The learning of cultural environment and cultural music can facilitate the people for their better psychological advancement [17]. Human emotions are related to their behavior, and people can improve their health when they have cultural associations. Music is considered the food of the soul, and it has relatable sentiments according to the living standards of the people that are motivating the people to deal with people better. Sometimes, there are negative and sometimes there are positive emotions influenced by music and culture. Human beings have different natures, and everyone has a different set of perceptions for their understanding [36]. However, every culture has a specific set of music, and the people belonging to this culture have a different set of mentality and culture. Access of people towards cultural information is possible through music. Indeed, people newly introduced to any culture also can get their critical information from their shared culture [23]. The accessibility to cultural understanding and information is improving the living standard of people concerning their cultural association. Music therapy is used almost in every culture for better human emotion development. The modern way of treatment is supported by music therapy, and based on this therapy people have relatable things to study [30]. The association of culture with music can’t be neglected, and it historically proved that every culture has a different set of music with relatable performances. Indeed, music performance and cultural awareness for music therapy are possible that can assist people to get a better understanding of their cultural values [21]. The relatable information to culture can promote cultural understanding for people and they can get a better approach towards sustainable psychology. Indeed, the role of music is critical in cultural awareness and promotion and people are required to focus on the cultural goals that are necessary for their better working approach. Music therapy is a widely used way of treatment in every culture, and people seem satisfied with this kind of treatment.

#### Hypothesis 1:

*There is a positive relationship between music therapy and sustainable psychological well-being.*

Health awareness is considered the fundamental way to improve the health status of the people. The people who are highly motivated to get health information, are seeking appropriate health opportunities in their life, and on the other hand, those who are not working to get better health facilities, are less motivated to get awareness [24]. The role of health awareness in the lives of people is critical because it can motivate them to get better treatment and work fairly. The relatable health information to the people and their cultural awareness can motivate them to get treatment for psychological well-being. People in every society have different sets of beliefs and opinions, and they are required to be motivated to a better standard of health and awareness [22]. The critical role of health awareness is necessary to improve the health standard of people, and people are required to have appropriate motivation to improve their psychological well-being. The role of music therapy is considered appropriate to improve human health, and people are required to get music therapy when they are not feeling well [10]. Almost, all people belonging to every set of age have a different set of opinions about their treatment. However, the better method to get emotional treatment is music therapy. By getting the entertainment of music, people can get into the catharsis that is necessary for their emotional improvement. The behavior of people for better achievement in health is possible when they have access to health awareness and better health standards [26]. The relatable health facilities are appropriately improved for the people when they are seeking any information. The awareness of the living standard of people is possible when they are highly motivated to get appropriate health standards. Reasonable health facilities are required for people to get better health opportunities, but music therapy should be used for both males and females to improve their health standards [8]. The way to improve public health can be a possible way to improve the standard of health in the right direction. People who are motivated to perform well, seek information that is appropriate to their better health standard. Reasonable working opportunities are required for people to get better health facilities when they are introduced to music therapy [19]. The influence of music therapy on public health is improved over time, and the awareness of the health facilities advanced people’s behavior for health awareness.

#### Hypothesis 2:

*There is a positive moderating role of health awareness between music therapy and sustainable psychological well-being.*

Maintaining health is a factor that is directly linked with people improving their work. Those individuals who are motivated to improve their health standards, are seeking relatable health awareness [18]. The advancement in health behavior can facilitate people to improve their health standards critically. Access to health is a significant factor that is improving public health facilities. Music therapy is considered one of the appropriate ways to promote public health. However, the people who are not motivated to get health facilities appropriately, are required to have health facilities in a significant way [9]. The relatable health facilities are appropriate for the public to improve their health standard accordingly. Awareness of health facilities can be a significant factor to improve the health behavior of the public. However, fewer people are motivated to get better health facilities, particularly music therapy in modern times. The awareness related to health facilities can promote the living standard of the public. In the meanwhile, those individuals who are less motivated to improve their health, are required to work appropriately for it [31]. Reasonable health facilities are appropriate for people, but they should have proper consciousness regarding their understanding and health facilities. The awareness to people of better health facilities can provide them with a way forward to improve their health reasonably. Furthermore, those people who have a systematic approach to improving their health, and they have rights plans for it, the health of these people is improved appropriately [33]. However, less focus on health improvement results in bad health conditions. Many people are highly motivated to improve the standard of their health, and they are required to improve their understanding and get treatment in new ways. The process of music therapy is simple in that people are motivated to improve the standard of their health with an effective approach. Access to public health can be a problem for people when they are motivated to improve their health standards [15]. Relatable health facilities are required for each disease, and for emotional disorders, therapy with music is highly recommended for the people. The advancement of modern treatment facilities is necessary for people to improve the standard of their health with effective performance and relatable opportunities. Music therapy is useful for people who are facing mental disorders or who are emotionally discouraged.

#### Hypothesis 3:

*There is a positive moderating role of strategic health management between music therapy and sustainable psychological well-being.*

**Fig. 1:**
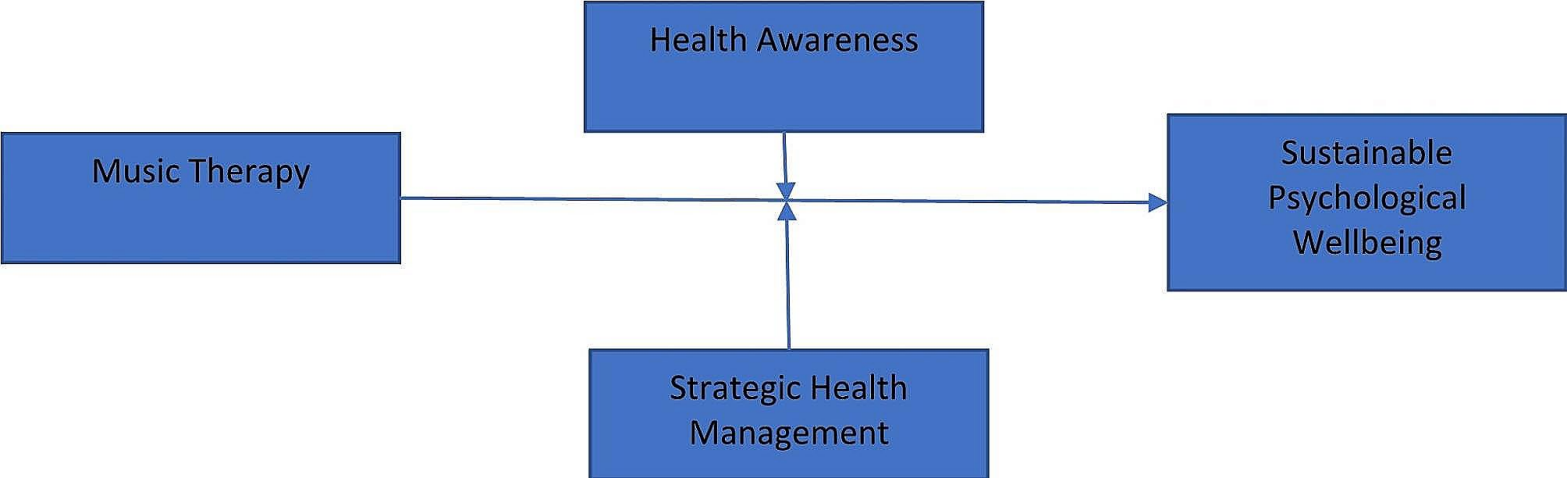
Theoretical model

The theoretical framework for this research is presented in Fig. 1.

## Methodology

### Questionnaire and pilot testing

In the current research, the questionnaire was developed by the experts to measure the relationship between numerous variables and test the hypotheses as a matter of results. In such way, in view of past understandable literature, the arrangement of the questionnaire was developed furthermore with the help of the associated concerns. However, the experts had to get on table, and share their viewpoints on different theories. The questionnaire was developed ourself.

This is confirmatory research in which the relationship between different variables is tested with empirical data. In this way, the research is based on the primary data directly collected from the respondents. Since, this study is discussing music therapy for Chinese people in general; therefore, this research population is based on the Chinese community in Mainland China. The study has considered the sample size as 342 because according to Morgan’s Table when the population of any research is more than 1,00,000, then the data collection from 384 respondents would be appropriate for research findings. There was no inclusion and exclusion criteria for the respondents because the population was selected generally. However, the study has considered a Likert scale questionnaire for data collection because this kind of questionnaire is widely used when the behavior and perceptions of the people are required to be measured. Furthermore, this research has considered the reflective questionnaire that can be measured easily by the data collected on the Likert scale questionnaire. The scale items for this research are taken from the findings of existing studies in the literature. Before considering the scale items from these studies, the reliability and validity of the scale items were also tested. In this way, this research has tested the findings of composite reliability > 0.70 and Cronbach alpha > 0.70 for significant findings. The scale items for music therapy are adapted from the study [37], and it was determined that the scale items have validity and reliability. In the same way, the scale items for sustainable psychological well-being are adapted from the study [14], and it was determined that the scale items have validity and reliability. Furthermore, the scale items for strategic health management are adapted from the study [38], and it was determined that the scale items have validity and reliability. Finally, the scale items for health awareness are adapted from the study [16], and it was determined that the scale items have validity and reliability. According to scholars, when coefficient of reliability such as Cronbach’s alpha is above 0.70 for scale items, the researchers can use same scale in future research. Hence, the reliability of all scales were established based on the findings of source studies. The developed questionnaire is reported in Appendix 1.

### Data collection procedure

The face validity of the scale items was also confirmed by the expert researchers, and it was ensured that the items have appropriate validity. The research used a cross-sectional method of data collection because the scale items were reflective in nature, and many social sciences studies also used a cross-sectional method for data collection and analysis. The duration of data collection was two months because of response rate. However, the respondents were first informed about the purpose of study before the collection of data. Furthermore, the questionnaires were distributed to 500 respondents, and 401 responses were collected back. The respondents were targeted randomly to collect the data. Out of 401, 384 responses were selected back for the final findings of this research. The study has determined the findings with Smart PLS 3.0, and it has utilized the findings of measurement model assessment including convergent validity and discriminant validity, and the findings of structural model assessment including the path findings. These findings are significantly considered for the final empirical evidence of this research support.

## Data analysis and findings

The collected data for this research is used to determine the missing values, skewness, and kurtosis. The findings of data normality are used to determine whether the data is useful or not. This research has considered the normality data test with skewness and kurtosis findings. The skewness values and the values of kurtosis for any research data are acceptable when lay between − 1 and + 1 [29]. The collected data for this research highlighted that the findings of this research have appropriate skewness and kurtosis values because no values were less than − 1 and more than + 1. In this way, the findings highlight that the study has the normality of data distribution. Furthermore, the analyzed data also showed the missing values, and there were no missing values in the data. Hence, the collected data for this research is appropriate and can be used for further tests. The findings are shown in Table 1.

**Table 1:** Normality of data

No.	Items	Missing	Mean	Standard deviation	Excess kurtosis	Skewness
1	MT1	0	4.082	1.056	0.821	-0.157
2	MT2	0	3.581	1.129	-0.217	-0.592
3	MT3	0	3.971	1.060	0.163	-0.867
4	MT4	0	3.95	1.011	0.215	-0.819
5	MT5	0	3.441	1.131	-0.422	-0.421
6	MT6	0	3.581	1.129	-0.458	-0.502
7	SPW1	0	3.584	1.200	-0.518	-0.556
8	SPW2	0	4.466	0.942	0.169	-0.908
9	SPW3	0	4.211	1.055	0.952	-0.297
10	SPW4	0	3.993	1.170	0.440	-0.120
11	SPW5	0	4.065	1.174	0.533	-0.194
12	SPW6	0	3.925	1.154	0.057	-0.936
13	SPW7	0	4.057	1.134	0.518	-0.150
14	HA1	0	4.118	1.086	0.900	-0.250
15	HA2	0	3.771	1.275	-0.419	-0.784
16	HA3	0	3.656	1.205	-0.459	-0.636
17	HA4	0	3.982	1.045	0.327	-0.910
18	HA5	0	4.039	1.003	0.553	-0.980
19	SHM1	0	4.004	1.032	0.721	-0.031
20	SHM2	0	4.036	1.057	0.654	-0.079
21	SHM3	0	3.961	1.121	0.454	-0.027
22	SHM4	0	3.781	1.126	0.059	-0.818
23	SHM5	0	3.86	1.129	0.260	-0.940

**Fig. 2:**
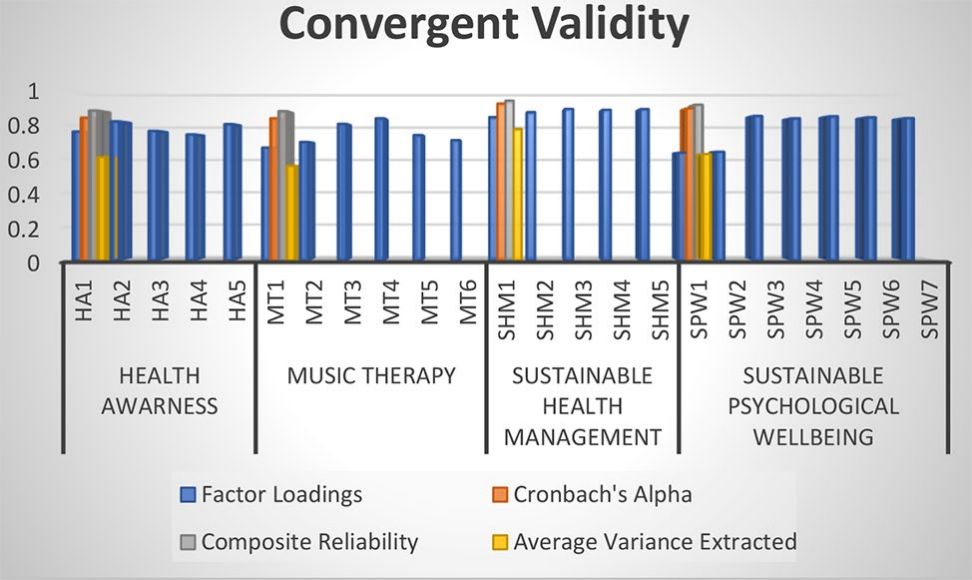
Convergent validity

This research also tested the findings of convergent validity to determine the reliability and validity of research data. The findings of Cronbach alpha, composite reliability, and average variance extracted are checked for it. The composite reliability is tested to determine the reliability of data, the Cronbach alpha is used to test the validity of data, and the average variance extracted is used to test the variance between research data. The significant Cronbach alpha is achieved when the values are more than 0.70 [32], the significant composite reliability is achieved when the findings are more than 0.70 [28], and the significant average variance extracted is achieved when the values are more than 0.50 [2]. The results of these three reported that the study has significant composite reliability > 0.70, significant Cronbach alpha > 0.50, and significant average variance extracted > 0.50. However, the findings of factor loadings were also tested, and the significant factor loadings are achieved when the values are more than 0.60. The analyzed values show that the factor loadings for each item were more than 0.60, and this research has reliable items for data collection. The results are shown in Table 2; Fig. 2.

**Table 2:** Convergent validity

Variables	Items	Factor loadings	Cronbach’s alpha	Composite reliability	Average variance extracted
Health Awareness	HA1	0.764	0.846	0.888	0.613
	HA2	0.824			
	HA3	0.768			
	HA4	0.747			
	HA5	0.808			
Music Therapy	MT1	0.669	0.842	0.884	0.561
	MT2	0.702			
	MT3	0.809			
	MT4	0.842			
	MT5	0.743			
	MT6	0.713			
Sustainable Health Management	SHM1	0.849	0.929	0.946	0.779
	SHM2	0.878			
	SHM3	0.897			
	SHM4	0.892			
	SHM5	0.896			
Sustainable Psychological Wellbeing	SPW1	0.638	0.900	0.922	0.631
	SPW2	0.644			
	SPW3	0.856			
	SPW4	0.842			
	SPW5	0.854			
	SPW6	0.848			
	SPW7	0.843			

**Fig. 3:**
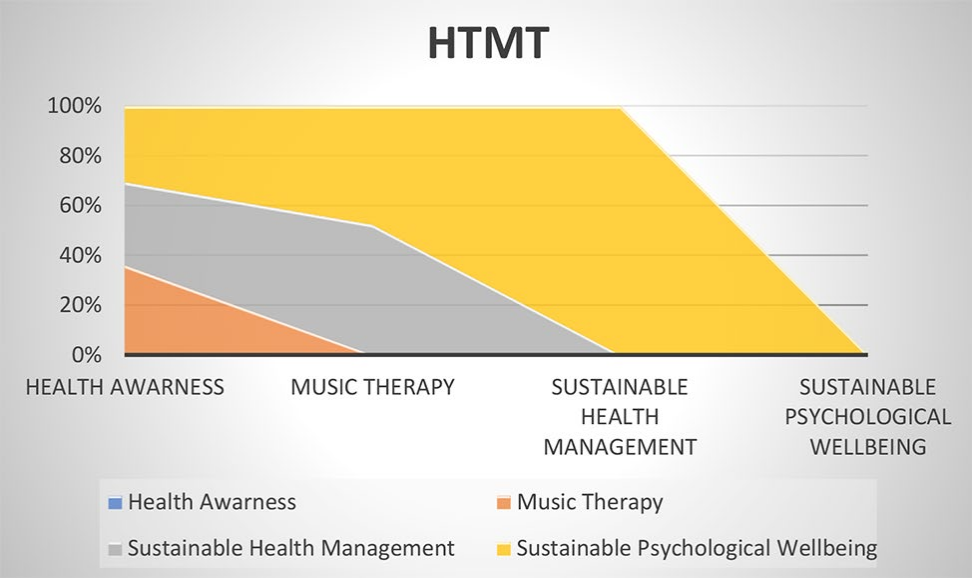
HTMT

The findings of discriminant validity are also tested to determine discrimination between the research data [13]. This research has used two methods for the determination of discriminant validity. The findings are tested with Heteritrait-Monotrait (HTMT) method earlier. This method is used by the data analysis technique of the social science studies. However, the findings of HTMT < 0.90 are significantly acceptable [11]. The reported data in Table 3; Fig. 3 showed that the appropriate HTMT is achieved by the research findings.

**Table 3:** HTMT

Variables	Health awareness	Music therapy	Sustainable health management	Sustainable psychological wellbeing
Health Awareness				
Music Therapy	0.782			
Sustainable Health Management	0.724	0.674		
Sustainable Psychological Wellbeing	0.664	0.615	0.546	

**Fig. 4:**
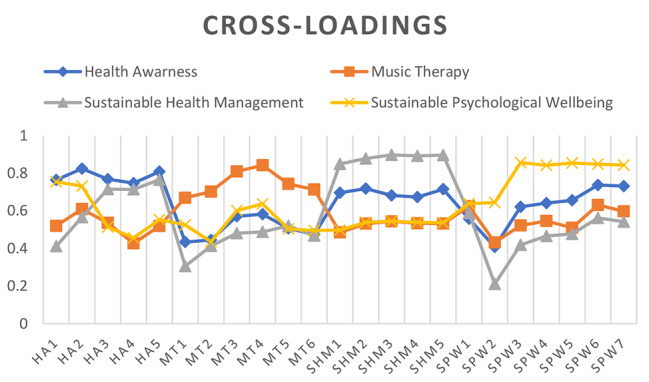
Cross-loadings

However, the findings of cross-loadings are also used to determine the discriminant validity. This method is used to test the discriminant validity between the research data at the individual item level. The findings of discriminant validity with cross-loadings are significant when the values of items representing one variable are greater than the values of items that are correlated with it [4]. The output data of the cross-loadings displayed in Fig. 4; Table 4 highlighted that this research has appropriately considered discriminant validity. Thus, the data for this research has appropriate discriminant validity and can be used significantly in future studies.

**Table 4:** Cross-loadings

Items	Health awareness	Music therapy	Sustainable health management	Sustainable psychological wellbeing
HA1	**0.764**	0.520	0.412	0.754
HA2	**0.824**	0.609	0.566	0.731
HA3	**0.768**	0.536	0.715	0.514
HA4	**0.747**	0.427	0.714	0.456
HA5	**0.808**	0.519	0.765	0.553
MT1	0.434	**0.669**	0.306	0.525
MT2	0.445	**0.702**	0.413	0.435
MT3	0.571	**0.809**	0.481	0.601
MT4	0.582	**0.842**	0.488	0.637
MT5	0.507	**0.743**	0.520	0.504
MT6	0.474	**0.713**	0.468	0.495
SHM1	0.696	0.485	**0.849**	0.498
SHM2	0.718	0.532	**0.878**	0.539
SHM3	0.682	0.544	**0.897**	0.545
SHM4	0.673	0.534	**0.892**	0.539
SHM5	0.715	0.532	**0.896**	0.537
SPW1	0.558	0.624	0.588	**0.638**
SPW2	0.408	0.432	0.211	**0.644**
SPW3	0.621	0.522	0.419	**0.856**
SPW4	0.641	0.546	0.466	**0.842**
SPW5	0.656	0.510	0.477	**0.854**
SPW6	0.737	0.631	0.562	**0.848**
SPW7	0.731	0.597	0.542	**0.843**

**Fig. 5:**
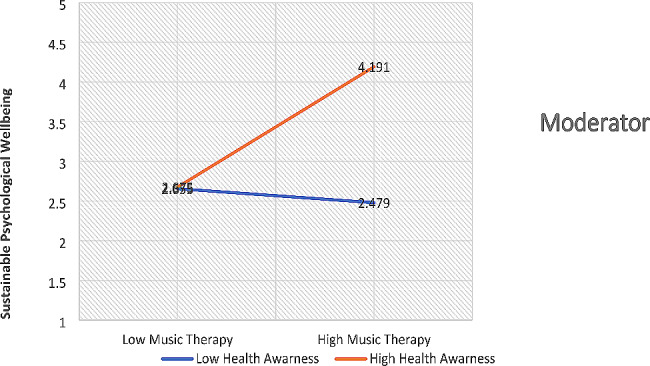
Moderation of health awareness

**Fig. 6:**
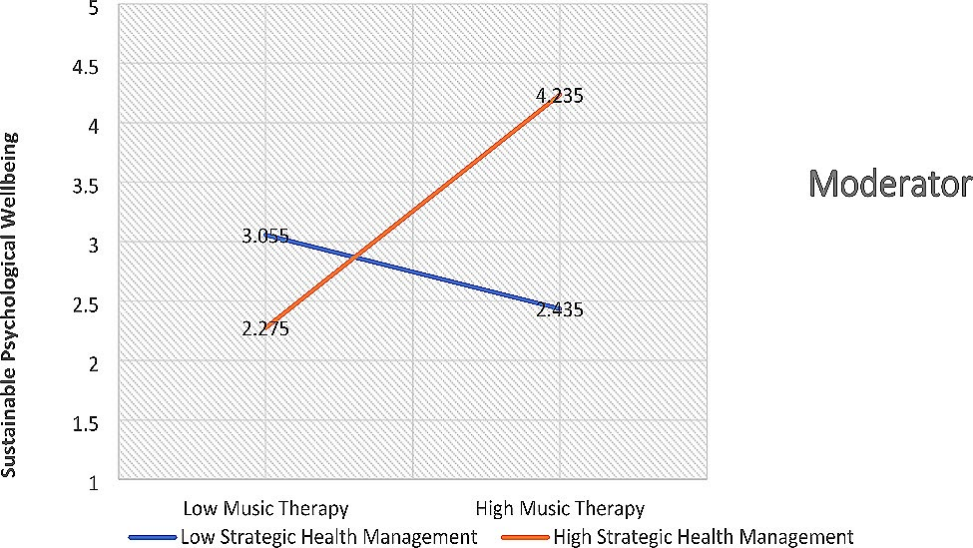
Strategic health management

**Fig. 7:**
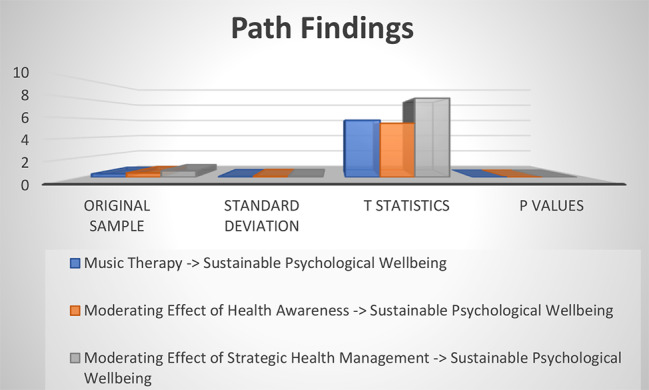
Path findings

Finally, the findings of the hypotheses were also tested, and this research has used structural equation modeling for path findings. The t-values were determined for the determination of the hypotheses’ status. The hypotheses of this research are directional, and the t > 1.64 is appropriate for significant hypotheses [27]. The analyzed data with the structural model reported that the first relationship of this research is significantly accepted, and there is a positive influence of music therapy on sustainable psychological well-being. The findings of the second hypothesis are also supported by the empirical data, and the study highlighted that there is a significant and positive moderating role of health awareness between music therapy and sustainable psychological well-being. This relationship is positive and strengthens the relationship between music therapy and sustainable psychological well-being, as shown in Fig. 5. Finally, the findings of the third hypothesis are also supported by the empirical data, and the study emphasized that there is a significant and positive moderating role of strategic health management between music therapy and sustainable psychological well-being. This relationship is positive and strengthens the relationship between music therapy and sustainable psychological well-being, as shown in Fig. 6. The results of structural equation modeling are highlighted in Fig. 7; Table 5.

**Table 5:** Path findings

Paths	Original sample	Standard deviation	T statistics	*P* values
Music Therapy -> Sustainable Psychological Wellbeing	0.335	0.058	5.805	0.000
Moderating Effect of Health Awareness -> Sustainable Psychological Wellbeing	0.423	0.077	5.493	0.000
Moderating Effect of Strategic Health Management -> Sustainable Psychological Wellbeing	0.645	0.080	8.036	0.000

## Discussion

The findings of this research are taken with measurement model assessment and structural model assessment. After the determination of data reliability, future tests are performed. The structural equation modeling approach is used to determine the findings of this research. The collected data significantly highlighted that the sustainable psychological well-being of the Chinese people is positively influenced by music therapy. Indeed, this relationship is newly developed in the literature, but the findings are in line with the conclusions of the existing studies in the literature. According to [10], music is regarded as the spirit’s nourishment since it evokes emotions that are relatable to people’s lifestyles and inspires them to interact with others more positively. The arts and entertainment may evoke negative emotions and other times good ones. People differ in nature, and each has a unique set of perceptions that they use to perceive things. According to [26], each culture has its distinct musical style and the individuals who make up that culture have their distinct mentalities and cultures. Music makes it feasible for people to access information about culture. A common language can indeed provide newcomers to any culture with essential knowledge. According to [15], one of the most important methods for enhancing human behavior and feelings is music therapy. While human behavior changes, it has a beneficial and important effect on the way people feel. Human behavior and psychology have advanced, which helps people function well in a variety of circumstances. People who struggle with their psychological well-being can experience a variety of cases, and they must work to improve their behavior and academic performance. According to [8], people’s improved psychological development can be aided by knowing about cultural environments and musical traditions. Human emotions and behavior are intertwined, and cultural associations help people enhance their overall well-being. Individual living standards are rising concerning their cultural affiliation thanks to the availability of cultural awareness and knowledge. Nearly every culture uses the use of music to promote the better development of feelings among individuals. According to [7], the application of music supports modern therapeutic methods, and patients can relate to the material studied as a result of this therapy. The public’s cultural awareness can be promoted by the knowledge that is relevant to them, and they can develop more effective strategies for practicing sustainability behavior. According to [9], music plays a crucial function in promoting understanding of cultures, and people must concentrate on the cultural objectives that are essential for their improved working methods. Every culture uses music therapy as a therapeutic method frequently, and people appear to enjoy it. According to [12], it is impossible to ignore how closely culture and music are related, and history has shown that each civilization has its distinctive musical repertoire and style of performance. It is feasible for people to learn more about their cultural values through performing musical pieces and cultural knowledge of music therapy. Hence, the role of music therapy should be considered practically to improve the psychological well-being of the public. It is important to advance the psychological well-being of the public which is helpful to improve. Therefore, the study rightly recommended the use of music therapy for improving psychological well-being of public.

Furthermore, this research has significantly reported that the empirical evidence supports that the moderating role of health awareness is significantly accepted between music therapy and sustainable psychological well-being. This relationship based on moderating effect is new in the literature on psychological well-being. However, the findings of this relationship are supported by the existing literature developed by the previous studies. According to [33], the primary means of enhancing people’s health status is thought to be raising their level of awareness of their health. On the contrary, people who have no opportunity to get superior medical care are less driven to get understanding. Individuals who are highly inspired to acquire health knowledge are looking for suitable healthcare possibilities throughout their lives. According to [36], the daily lives of individuals depend heavily on health knowledge since it can inspire them to receive better care and treat others decently. People may be encouraged to seek therapy for mental well-being if their medical data is relevant to them and they are aware of cultural differences. Individuals can enter the healing process that is required for psychological development by listening to interesting music. According to [17], whenever individuals have accessibility to improved health standards and health understanding, they are more likely to act in ways that will improve their health outcomes. When people are looking for information, the relevant healthcare facilities are upgraded appropriately. People can become conscious of their living standards when they are very motivated to achieve the right level of health. According to [22], while sufficient medical services are necessary for people to have better health opportunities, both men and women should use therapy in music to raise their standard of health. When someone is ill, they must take lessons in music therapy because music therapy is thought to be a useful tool for enhancing human health. According to [5], regardless of age, has a different set of thoughts about how they should be treated. However, music therapy is a more effective way to treat emotional issues. A potential strategy for raising the standard of health in the right direction is to enhance the health of the population. Individuals who are driven to succeed look to find knowledge that is compatible with their higher standard of health. According to [35], when people are introduced to music therapy, reasonable employment opportunities are necessary for them to have access to better healthcare. Each member of society holds a unique set of beliefs and perspectives, and it is necessary to inspire them to strive for a higher level of knowledge and health. Individuals must have the proper incentive to improve their mental happiness, and health knowledge plays a crucial part in raising people’s health standards. According to [23], the impact of music therapy on the general public has grown throughout time, and the public’s understanding of health resources has advanced their behavior in terms of health consciousness. The role of health awareness is reliable for the improvement in health standards of the public. Hence, the practitioners are motivated to use the music theory for psychological well-being of the public by ensuring the health awareness in public. The health awareness can provide better and reliable opportunities for the public to improve their standards of health.

Lastly, this research has significantly reported that the empirical evidence supports that the moderating role of strategic health management is significantly accepted between music therapy and sustainable psychological well-being. No doubt, this relationship based on moderating effect is new in the literature on psychological well-being. Nevertheless, the findings of this relationship are supported by the existing literature developed by the previous studies. According to [21], the component that is directly related to people’s ability to work better is the maintenance of their health. People who are motivated to raise their standard of health are looking for relatable health information. The development of healthy behaviors can help people significantly raise their level of health. The public’s medical facilities are being improved in large part due to increased access to healthcare. One of the relevant strategies for promoting public health is thought to be music therapy. According to [24], those who lack the necessary motivation to obtain medical treatment must be forced to do so in a significant manner. The required health facilities are available for the general public to raise their current level of health. Consumers can go forward to enhance their health practically by being more aware of improved medical resources. According to [31], the physical well-being of these individuals has enhanced appropriately if they have an organized method to doing so and the proper plans in place. However, failing to prioritize health improvement leads to poor health. Many people have a strong desire to raise their quality of health, but they must also learn more and receive care through novel methods. People who have the urge to better their health can do so through the straightforward process of therapeutic music listening. People’s knowledge about medical care can raise their level of living. According to [30], those people who are less driven to enhance their health must work appropriately for it in the interim. Individuals ought to possess proper awareness of their comprehension and medical facilities to benefit from reasonable healthcare resources. When someone is determined to raise their quality of health, getting access to healthcare services can be a challenge. According to [39], especially for each ailment, appropriate medical facilities are needed, and for persons with emotional disorders, music therapy is strongly advised. Health-related behavior can be significantly improved by raising awareness about medical resources. Nevertheless, fewer people today have the desire to access better healthcare, especially when it comes to the area of music therapy. For individuals to maintain their level of health with successful outcomes and relevant chances, cutting-edge medical services must advance. According to [18], people with mental illnesses or those who are emotionally depressed can benefit from learning about music therapy. Practically, this study also recommended that the health management should be in a strategic way by the people. It is important because strategic health management provides a way forward to the public for improving their psychological well-being. Hence, the findings of this research are critically important for advancement in health standards.

## Conclusion

To sum up, the current study has some theoretical and practical implications to advance the literature and practice for sustainable psychological well-being respectively in post-covid 19 era. In clinical practice in post pandemic period, music therapy can be effectively used to improve the psychological well-being of individuals with sustainability. The information related to health awareness and practice for strategic health management is also necessary for the clinical patients to improve their psychological well-being. To conclude, the study asserted the importance of music therapy for improvement in psychological well-being of the Chinese community in post covid-19 era. The study recommends that the psychological well-being of the people was disturbed in post-covid-19 era which needs appropriate improvement for their better social life. The clinical practitioners are required to work in this direction to improve the level of their learning and performance.

### Theoretical and practical implications

This research has developed a newly considered relationship based on theoretical underpinning. However, the empirical results supported the theoretically developed relationship of this research after post-covid era. The research advanced the literature of post covid-19 era on psychological well-being and reported that there is a significant and positive impact of music therapy on the sustainable psychological well-being of students. This relationship has introduced a new factor that is influencing sustainable psychological well-being. Furthermore, this study has contributed two moderating variables between the relationship between music therapy and sustainable psychological well-being, and the literature on psychological well-being is advanced by this research. The study has reported that health awareness is a significant moderator between music therapy and sustainable psychological well-being. This newly developed relationship also advanced the knowledge of psychological well-being with a significant moderating influence. Finally, the study has reported that strategic health management is a significant moderator between music therapy and sustainable psychological well-being. This newly developed relationship also advanced the knowledge of psychological well-being with a significant moderating influence. These findings also advanced the therapy of Psychodynamics as it is significantly accepted in the relationship between music therapy and sustainable psychological well-being. This research has novel findings as it has comprehensively introduced that the relationship between music therapy and psychological well-being can be strong when there is moderating influence of health awareness and strategic health management.

This research also has practical implications based on its significant findings. The study reported that music therapy should be considered the appropriate method to improve the psychological well-being of people. Indeed, people should go for music therapy when they are not appropriately treated for their emotional disorders or mental problems. Emotional health issues are critical, and these issues can damage the public who has a negative emotional state. However, the concerns for emotions are required to be improved with better psychological advancement. The required psychological improvement with music therapy can improve the health awareness and attitude of the public. The clinical treatment should add music therapy as a significant method of emotional treatment for those who are not in good mental health. This study also demonstrated that health awareness is necessary for people to get music therapy. The people have emotions and sentiments, but they can get better and more effective ways of health awareness when the government is creating awareness of health. The healthcare departments are required to promote health awareness in a critical way that should be acceptable for the public to improve their critical performance. However, strategic health management is also required to improve the health awareness of people which can be a significant factor in the advancement of health facilities for the public. The critical role of health awareness programs is to improve the health standard of the public with the newly introduced treatment of music therapy. The mental health of people can be improved significantly with music therapy. The clinical practitioners for psychological well-being can work on these practice-based recommendations to use music therapy for advancement in psychological well-being of the community. The role of music theory and health awareness is considered as critical in this research for advancement in psychological well-being of the community.

### Future directions

No doubt, the findings of the study reported that there is a significant and positive influence of music therapy on the sustainable psychological well-being of the Chinese community. On the other hand, this study also concluded that there is a significant and positive moderating role of health awareness and strategic health management between music therapy and sustainable psychological well-being. The findings of this research are new and novel in the literature on psychological well-being. This research has some theoretical and practical implications to advance the literature and practice for sustainable psychological well-being respectively. Similar to the other studies, this research also has some limitations that are required to be addressed by future studies. This research has limitations as it has collected data from the Chinese population only, and the findings of this research can’t be generalized because data collection from backward countries may not support these results as people don’t have access to music therapy. Therefore, scholars are required to collect data from the population of another country to provide significant findings in the literature. Moreover, this research has collected data with a cross-sectional approach which is its limitation. In this way, the scholars are motivated to use the longitudinal data in future studies to understand the consequences of music therapy as well in post covid-19 era. Hence, it would be a significant addition to the body of knowledge.

### Electronic supplementary material

Below is the link to the electronic supplementary material.

Supplementary Material 1

## Data Availability

All the data within the manuscript.
