# Supplementary material for: Psychological wellbeing with music therapy: the moderating role of health awareness, and strategic health management in post Covid-19 era
Source: BMC Psychol. 2024 Jun 18;12:355. doi: 10.1186/s40359-024-01845-z (PMC11184797; doi:10.1186/s40359-024-01845-z)
Supplement: Supplementary file 1 — Supplementary Material 1 [file 40359_2024_1845_MOESM1_ESM.docx]

**Questionnaire**

| Variables | Items |
| --- | --- |
| Health Awareness | I go for clinical checkup when feel ill. |
|  | The health awareness is important to improve health. |
|  | I have appropriate information about my health. |
|  | I believe information about health is necessary for everyone. |
|  | I attend seminars for understanding health awareness. |
| Music Therapy | The music therapy is good for mental health. |
|  | Emotional health is improved with music therapy. |
|  | I believe music therapy as source of psychological improvement. |
|  | I recommend music therapy for better health management. |
|  | I understand music therapy is important to reduce the chances of nervous breakdown. |
|  | I trust on the importance of music therapy. |
|  | I am a health-conscious person. |
| Sustainable | I believe health management is important for better life. |
| Health | I visit clinic regularly to get clinical treatment. |
| Management | Health management has become a factor for improving my health. |
|  | Sustainability in health is necessary for better mental health. |
|  | I am emotionally strong and have good mental health. |
|  | I have maximum psychological control on me. |
| Sustainable | I feel emotionally strong in different situations. |
| Psychological | I improve my mental health which is good for my health. |
| Wellbeing | I take psychological therapies for better mental health. |
|  | I am emotionally strong by nature. |
|  | I believe psychological problems are caused by mental health. |
